# Exploring strategies for trachoma elimination in Ethiopia

**Published:** 2022-01-31

**Authors:** Adugna Amin, Alemayehu Sisay, Tim Jesudason

**Affiliations:** 1 NTDs Programme Manager: Light for the World, Addis Ababa, Ethiopia.; 2 Country Director: Orbis Ethiopia, Addis Ababa, Ethiopia.; 3 Special Projects and Campaign Partnerships: International Coalition for Trachoma Control.

**Human resources for trachoma elimination remain a challenge in the remote communities of Ethiopia.**

**Figure:**
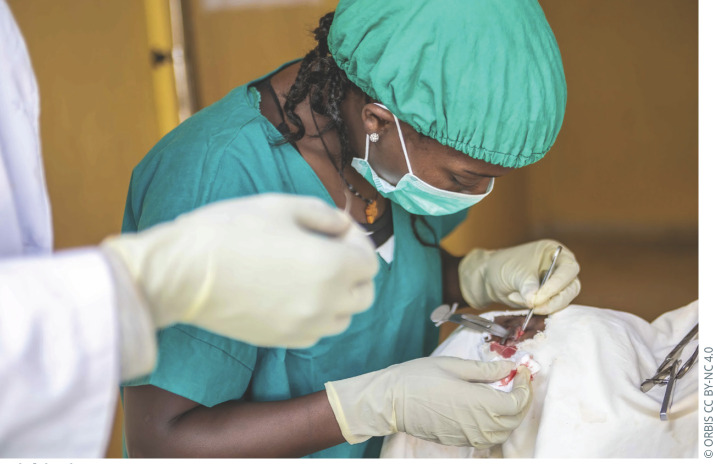
Trichiasis surgery. **ETHIOPIA**

Ethiopia has the greatest burden of trachoma worldwide, accounting for 49% of 136.2 million people at risk globally.^1^ In 2021, the World Health Organization (WHO) reported that almost 460,000 people in Ethiopia require surgery to treat trachomatous trichiasis (TT), the late blinding stage of the disease.^1^

Although the Ethiopian Federal Ministry of Health has made significant progress in scaling up all components of the WHO-endorsed SAFE strategy (surgery, antibiotics, facial cleanliness, and environmental improvement), efforts to reduce the TT backlog are complicated by the remoteness of the communities affected by the disease, limited access to health services, and inadequate human resources for eye health to provide standardised TT surgery training and high quality treatment.

To maximise the impact of its national trachoma programme, the Ethiopian Federal Ministry of Health has implemented two human resource strategies across eye health services and the trachoma programme to progress towards trachoma elimination targets. One strategy trains general health workers, and the other trains allied eye health workers.

## 1. General health workers

In several parts of the country, including the regions of Amhara, Oromia, and SNNP, the backlog is being addressed by the training, certification, and supervision of general health workers, including general nurses, to identify and manage TT, including by providing TT surgery. In Ethiopia, this group of health workers is known as integrated eye care workers (IECWs); they work at the primary health care level.

This model requires the empowerment of local authorities to provide supervision as well as technical and administrative support. By upskilling existing human resources, it has been successful in scaling up case-finding and management of TT in the regions where it is being implemented.

However, several challenges remain. As TT cases decline, the quality of surgery becomes difficult to maintain since IECWs conduct fewer operations and thereby have less opportunity to practise their skills. It has also been observed that IECWs have much higher rates of turnover due to the lack of career pathways and limited incentives. This can increase the costs of training and have a negative impact on the quality of care.^2^

## 2. Allied eye health workers

An alternative strategy is being used in the region of Tigray, where allied eye health workers (ophthalmic nurses, ophthalmic officers, and cataract surgeons) are trained and deployed to clear the TT backlog in the region. Ethiopia’s national trachoma programme trains and certifies these allied eye health workers to carry out TT surgery as per the WHO Trichiasis surgery for trachoma manual (**bit.ly/TT-surgery**).

Upskilling ophthalmic nurses and ophthalmic officers has several benefits. First, during TT surgery campaigns, the use of specialist eye care workers means patients with other eye conditions can be treated or referred to secondary level eye care units when diagnosed with conditions such as cataract or glaucoma. The use of allied eye health workers therefore better facilitates the transition of the trachoma programme into the existing eye health system. This helps to ensure high quality of care and improves trust in services.

*Ending the neglect to attain the Sustainable Development Goals: A road map for neglected tropical diseases 2021–2030* (**bit.ly/WHOsdg**) and the WHO *World Report on Vision* (**bit.ly/wrvision)** recognise that resolving human resource challenges for eye health will be critical to achieving and sustaining the elimination of trachoma as a public health problem. In the short term, strategies such as training general health workers provide countries with high burdens an opportunity to reduce the backlog of TT cases. However, to ensure the sustainability and quality of programs, it is critical that TT services are integrated within eye health systems and delivered by well trained and supervised eye care professionals who have a clear career track and opportunities for growth. Achieving this will require political will and domestic financing to integrate trachoma interventions into national eye health programmes.
